# EBV-positive inflammatory follicular dendritic cell sarcoma occurring in different organs: a case report and literature review

**DOI:** 10.3389/fonc.2025.1603496

**Published:** 2025-07-31

**Authors:** Wenhua Bai, Chunfang Hu, Zheng Zhu

**Affiliations:** ^1^ Department of Diagnostic Radiology, National Cancer Center/National Clinical Research Center for Cancer/Cancer Hospital, Chinese Academy of Medical Sciences and Peking Union Medical College, Beijing, China; ^2^ Department of Pathology, National Cancer Center/National Clinical Research Center for Cancer/Cancer Hospital, Chinese Academy of Medical Sciences and Peking Union Medical College, Beijing, China

**Keywords:** EBV-positive inflammatory follicular dendritic cell sarcoma, Epstein-Barr virus, computed tomography, magnetic resonance imaging, case report

## Abstract

**Background:**

Epstein-Barr virus (EBV)-positive inflammatory follicular dendritic cell sarcoma (EBV+ IFDCS) represents a low-grade malignancy arising from the proliferation of follicular dendritic cells. This distinct and rare subtype, characterized by abundant lymphoplasmacytic infiltration, is closely linked to EBV infection and is seldom encountered in clinical practice.

**Methods:**

Presented here are three cases of primary EBV+ IFDCS, occurring in the liver and spleen. This study systematically analyzed the clinical presentations, radiological features, and pathological characteristics of our cases. Additionally, we conducted a comprehensive review of the respective characteristics documented in the existing literature.

**Results:**

We present three cases of EBV+ IFDCS, with lesions localized to the spleen (n=2) and liver (n=1). Notably, only one patient developed clinical symptoms secondary to splenic mass rupture and post-embolization sequelae, while the remaining cases were identified incidentally without associated symptomatology. All three patients underwent preoperative contrast-enhanced magnetic resonance imaging (CT) scans demonstrating solitary, well-circumscribed round masses/nodules. The two splenic lesions exhibited necrotic-cystic degeneration and one displayed a capsule, with absence of calcification in all cases. Tumor parenchyma showed mild arterial-phase enhancement and partial delayed-phase washout. The two splenic cases underwent additional magnetic resonance imaging (MRI) evaluation, revealing restricted diffusion in the solid tumor components and apparent diffusion coefficient (ADC) values comparable to the surrounding splenic parenchyma. Complete surgical excision was performed in all patients, and histopathological evaluation confirmed the diagnosis of EBV+ IFDCS through immunohistochemical analysis. As of the latest follow-up, all three patients are alive.

**Conclusion:**

EBV+ IFDCS is a rare condition that primarily arises in the liver and spleen, with prognosis varying among patients with primary tumors in different organs. This study presents three cases of EBV+ IFDCS that occurred in diverse anatomical locations, examines their clinical, radiological, pathological features and differential diagnoses, and aims to deepen the understanding of clinicians and radiologists regarding this form of Mesenchymal dendritic cell neoplasm.

## Introduction

EBV-positive inflammatory follicular dendritic cell sarcoma (EBV+ IFDCS) is an exceptionally rare low-grade malignant tumor. According to World Health Organization (WHO) Classification of Tumors of Haematopoietic and Lymphoid Tissues, 5th edition (WHO-HAEM5), it is classified as a distinct subtype under “Mesenchymal dendritic cell neoplasm”, with particular emphasis on its “inflammatory” characteristics (a background of lymphoplasmacytic infiltration) and the central role of EBV infection in its pathogenesis (1). This distinguishes it from traditional non-inflammatory follicular dendritic cell sarcomas. Globally, its annual incidence remains undefined due to its extreme rarity and frequent misdiagnosis. The tumor predominantly occurs in the liver and spleen, while involvement of the lungs, gastrointestinal tract, or lymph nodes is exceedingly uncommon (2, 3).

The three cases reported herein - spanning the spleen and liver - expand the documented spectrum of EBV+ IFDCS and reinforce the centrality of EBV in its pathogenesis.

## Case presentation

### Case 1

**Figure 1:**
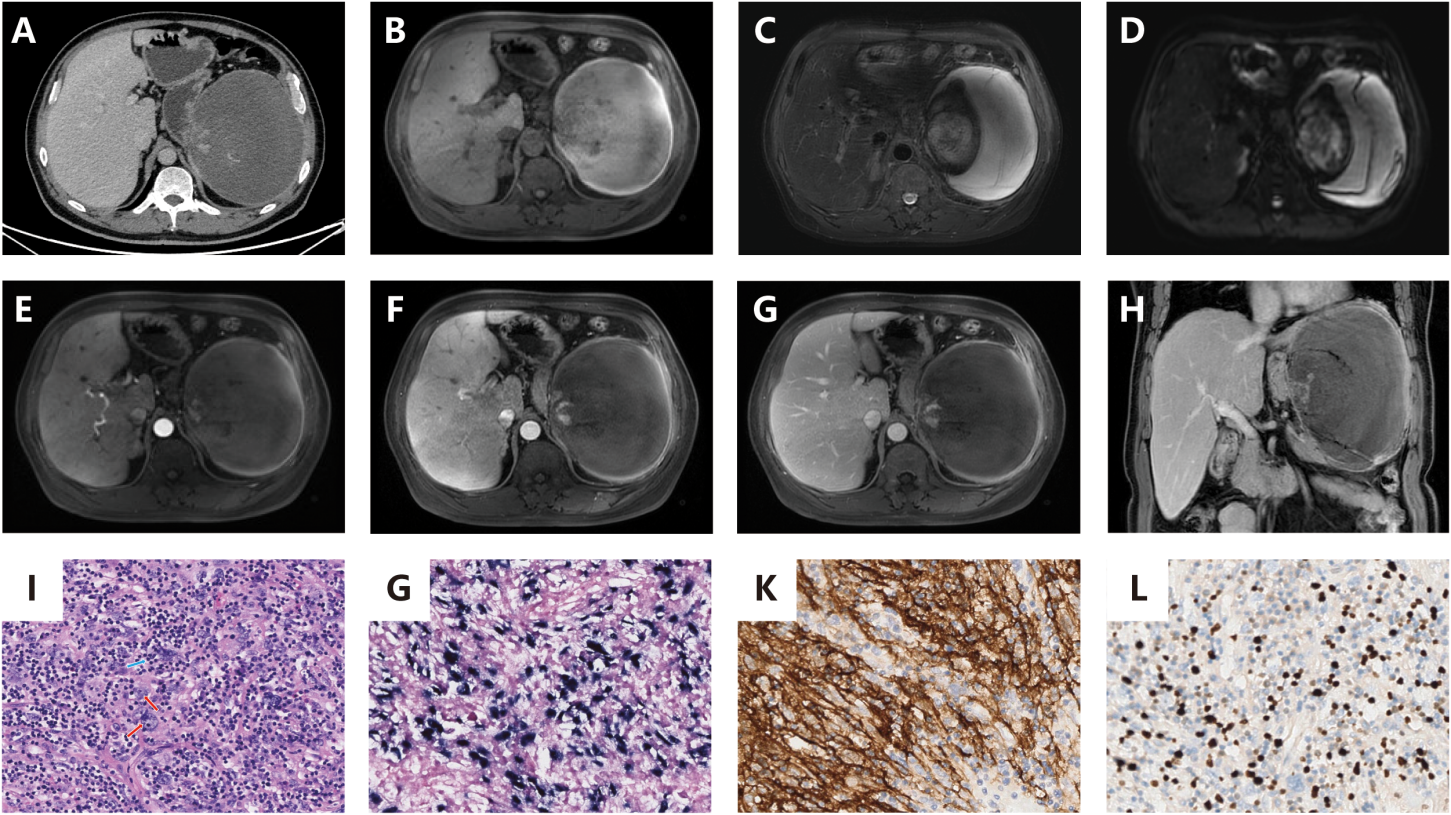
**(A)**: Contrast-enhanced CT reveals a well-defined, rounded splenic mass with mild enhancement of solid components, a visible capsule, absence of calcification, and features of hemorrhage and necrotic cystic degeneration. No evidence of metastatic spread was identified; **(B)**: Contrast-enhanced MRI demonstrates a heterogeneously hypointense signal on pre-contrast T1-weighted imaging; **(C)**: T2-weighted MRI sequences show heterogeneously intermediate-to-hyperintense signal with internal hypointense areas; **(D)**: DWI reveals heterogeneously hyperintense signal; **(E–G)** (axial): Post-contrast axial scans exhibit minimal enhancement in most regions with focal enhancing areas, showing no delayed phase progression. The tumor is encapsulated and devoid of fatty components; **(H)** (coronal): Post-contrast coronal scan confirms the encapsulated tumor with minimal and focal enhancement; **(I)** (x200): Histopathological examination with hematoxylin-eosin (HE) staining displays spindle-shaped neoplastic cells arranged in a storiform pattern, featuring indistinct cellular boundaries, prominent nucleoli, and marked inflammatory infiltrate (red arrow: neoplastic cells; blue arrow: lymphocytes); **(J)** (x200): EBER *in situ* hybridization confirms nuclear positivity within tumor cells; **(K)** (x200): Immunohistochemical analysis reveals CD21 membranous expression in neoplastic cells; **(L)** (x200): Ki-67 labeling demonstrates a low proliferative index.

The 54-year-old male patient was referred to our hospital after a splenic puncture rupture and subsequent splenic embolization. The patient had no significant medical or family history of similar conditions. Imaging at our facility showed a well-defined, roundish mass in the spleen with heterogeneous density on computed tomography (CT) and mixed signal intensity on magnetic resonance imaging (MRI), indicative of necrosis and hemorrhage, without evidence of metastasis (**Figure 1**).

The patient underwent a splenectomy. Intraoperative findings included an enlarged, irregular spleen without significant adhesions. Pathological examination of the resected spleen revealed a spindle cell tumor with extensive necrosis and lymphoplasmacytic infiltration, measuring 25×17×6 cm overall, with the solid portion being 10×7×6.5 cm. Immunohistochemical analysis confirmed the presence of CD163, CD21, and EBER positivity, leading to a diagnosis of EBV+ IFDCS.

Postoperatively, the patient recovered well without complications. At the last follow-up on March 17, 2025, there were no signs of recurrence.

### Case 2

**Figure 2:**
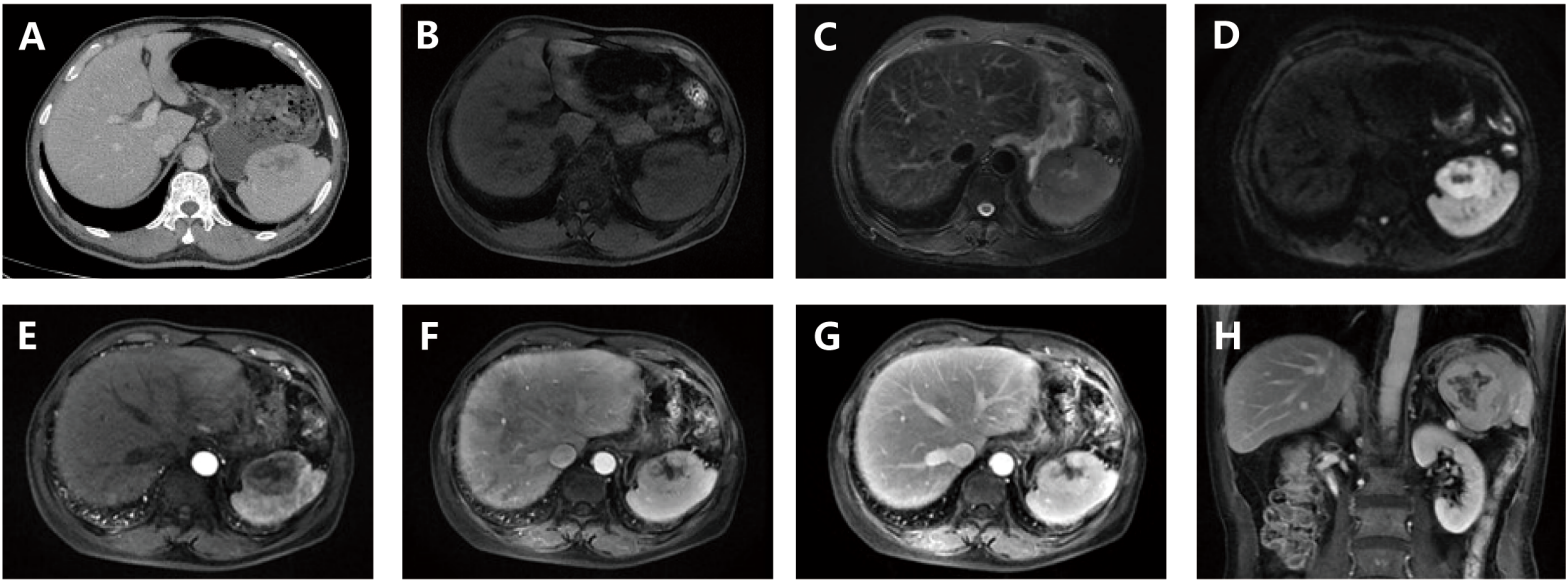
**(A)** Contrast-enhanced CT demonstrates a splenic space-occupying lesion (mass) exhibiting a rounded morphology with ill-defined margins. The solid components display mild contrast enhancement, while cystic necrosis and poorly-defined hemorrhage are observed. Absence of a capsule, calcification, or metastatic features is noted; **(B)** Pre-contrast MRI shows the lesion with peripheral isointensity and central hypointensity; **(C)** T2-weighted MRI demonstrates peripheral isointensity with central intermediate-to-hyperintense signal; **(D)** DWI) reveals peripheral hyperintensity surrounding a central hypointense region; **(E–G)** (axial): Post-contrast axial imaging reveals mild-to-moderate heterogeneous enhancement without evidence of encapsulation or fatty components; **(H)** (coronal): Post-contrast coronal imaging confirms mild-to-moderate heterogeneous enhancement without evidence of encapsulation or fatty components.

A 65-year-old male with a history of gastric cancer was admitted for evaluation of a splenic mass found during routine follow-up. He showed no symptoms and there was no family history of similar conditions. Imaging revealed a poorly defined, roundish mass in the spleen with mild enhancement on CT and mixed signal intensity on MRI, indicating necrosis with chronic hemorrhage but no evidence of a capsule, calcification, or metastasis (**Figure 2**).

The patient underwent a splenectomy. Intraoperatively, an enlarged, irregular spleen without significant adhesions was noted. The resected spleen measured 7.5×6×5 cm, containing a 5.5×4.6×4 cm gray-white, firm tumor with central hemorrhage near the capsule. Pathology diagnosed spindle cell proliferation with lymphoplasmacytic infiltration. Immunohistochemistry confirmed positivity for SMA, Vimentin, Ki-67 (15%), and follicular dendritic cell markers (CD21, CD23, CD35), along with EBER positivity.

The final diagnosis was EBV+ IFDCS. Post-splenectomy, the patient recovered well without complications. At the last follow-up on March 17, 2025, there were no signs of recurrence.

### Case 3

**Figure 3:**
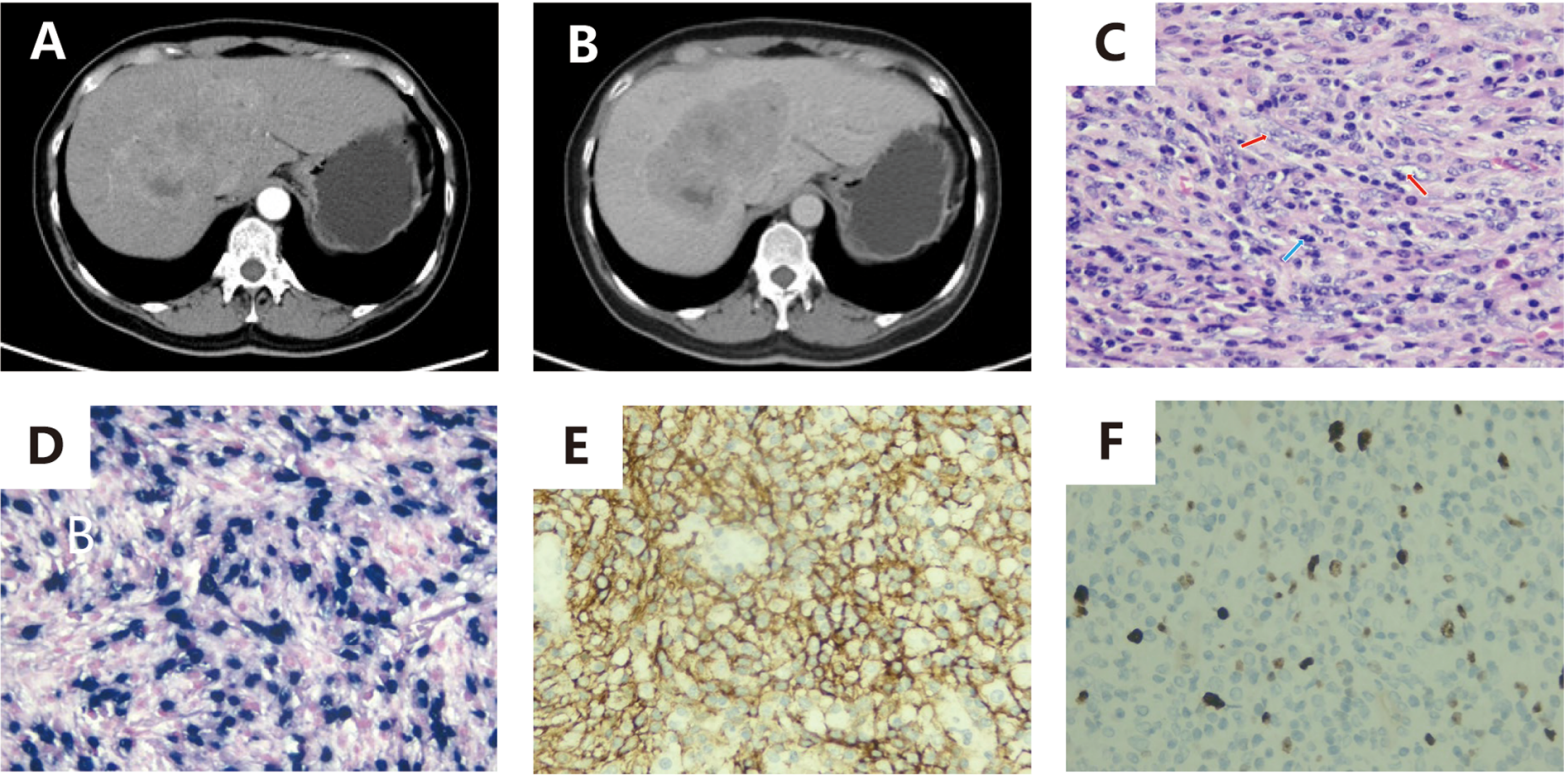
**(A, B)**: Contrast-enhanced CT demonstrates an irregularly shaped, poorly demarcated space-occupying lesion within the hepatic hilar region. The lesion exhibits progressive mild-to-moderate enhancement on post-contrast phases, accompanied by internal cystic necrosis. Absence of encapsulation, calcification, hemorrhage, or metastatic features is noted; **(C)** (x200): Histopathological evaluation with HE staining displays neoplastic cells arranged in a storiform pattern, characterized by plump spindle-shaped morphology, indistinct cellular boundaries, prominent nucleoli, pale cytoplasm, and marked inflammatory infiltrate (red arrow: neoplastic cells; blue arrow: lymphocytes); **(D)** (x200): EBER *in situ* hybridization confirms nuclear positivity within tumor cells; **(E)** (x200): Immunohistochemical analysis reveals CD21 membranous expression in neoplastic cells; **F** (x200): Ki-67 labeling demonstrates a low proliferative index.

A 53-year-old female was admitted for a hepatic mass found in the liver. She had previously undergone three cycles of chemotherapy and had no significant symptoms or family history of similar conditions. Imaging revealed an ill-defined, irregular mass in the liver’s hilar region with low-density on CT and mild-to-moderate enhancement, showing necrotic changes but no capsule, calcification, hemorrhage, or metastasis (**Figure 3**).

She underwent a middle hepatectomy. The resected specimen contained a 13×11×6.5 cm gray-white, firm tumor with areas of necrosis. Pathology diagnosed spindle or ovoid cell proliferation with lymphocytic and plasmacytic infiltration, and immunohistochemistry confirmed EBV+ IFDCS.

The final diagnosis was EBV+ IFDCS. Postoperatively, she recovered well without complications. The most recent follow-up occurred on March 17, 2025, at which time the patient was alive with the tumor still present. During the period following the surgery and up to the last follow-up, there was a recurrence of the tumor within the liver. Currently, this recurrence is being managed through ablation therapy.

## Discussion

This study provides the analysis of EBV+ IFDCS involving two distinct anatomical sites (spleen, liver), elucidating the organ-specific radiological-pathological correlations and diagnostic challenges within the WHO 5th edition classification framework. According to the WHO-HAEM5 released in 2022, EBV+ IFDCS is formally defined as a distinct stromal-derived dendritic cell tumor (1). As a distinct disease entity, the fifth edition further clarifies the biological distinctions between EBV+ IFDCS and conventional FDCS, while highlighting the central role of EBV in its pathogenesis (1, 4).

EBV+ IFDCS primarily occurs in the spleen and liver and exhibits relatively distinct characteristics, such as a female predominance, prolonged clinical course, presence of a prominent inflammatory background, and association with EBV infection. In our analysis of previously published literature, we identified a total of 35 cases of EBV+ IFDCS in the spleen (5–20), and 30 cases in the liver (8, 16, 17, 21–38), including the case presented in this report (Spleen: NO.34 and 35; Liver: NO.30) (**Table 1**). Our statistical findings indicate that both splenic and hepatic EBV+ IFDCS cases predominantly affect females, with a male-to-female ratio of approximately 1:1.82, aligning with prior studies. Follow-up data spanning between 2 and 108 months were available for 59 patients. Among the 29 patients with splenic EBV+ IFDCS who had follow-up data, only one death was reported by the last follow-up, with no observed recurrences, yielding an overall mortality rate of 3.4%. Conversely, among the 30 patients with hepatic IFDCS who had follow-up data, two deaths and four recurrences were noted by the last follow-up, resulting in an overall mortality rate of 6.7% and a recurrence rate of 13.3%.

**Table 1:** Literature review of EBV-associated FDCS of spleen and liver.

Location	Case no.	Sex	Age (y)	Maximum diameter^*^ (cm)	Treatment	Follow-up (months)	Outcome	Reference
Spleen	1	F	63	15	Splenectomy	NA	NA	Lennert (5)
	2	F	70	3	Splenectomy	NA	NA	Monda et al. (6)
	3	F	54	3.5	Splenectomy	10	No recurrence	Fabio et al. (7)
	4	M	79	6	Splenectomy	18	No recurrence	Fabio et al. (7)
	5	M	29	11	Splenectomy	12	No recurrence	Cheuk et al. (8)
	6	M	59	9.5	Splenectomy	17	No recurrence	Leng, Yu, and Wang (9)
	7	F	64	7.2	Splenectomy	8	No recurrence	Pagliuca et al. (10)
	8	M	61	6.2	Splenectomy	16	No recurrence	Pagliuca et al. (10)
	9	F	42	4	Splenectomy	9	No recurrence	Pagliuca et al. (10)
	10	F	57	13.3	Splenectomy	4	Alive	Pagliuca et al. (10)
	11	M	52	3.7	Splenectomy	5	Alive	Pagliuca et al. (10)
	12	F	57	5	Splenectomy	12	No recurrence	Mograbi et al. (11)
	13	F	59	4.5	Splenectomy	NA	NA	Ge et al. (12)
	14	M	57	2.7	Splenectomy	9	No recurrence	Zhang et al. (13)
	15	F	64	5.5	Splenectomy	78	No recurrence	Li et al. (14)
	16	F	72	7.2	Splenectomy	18	No recurrence	Li et al. (14)
	17	F	53	3.2	Splenectomy	13	No recurrence	Li et al. (14)
	18	M	76	3.2	Splenectomy	8	No recurrence	Li et al. (14)
	19	M	72	6	Splenectomy	18	No recurrence	Li et al. (14)
	20	M	75	3.5	Splenectomy	30	No recurrence	Li et al. (14)
	21	F	69	6	Splenectomy	NA	NA	Morales-Vargas et al. (15)
	22	F	66	5	Splenectomy	6	No recurrence	Yanyang et al. (16)
	23	M	39	7.4	Splenectomy	40	No recurrence	Kazemimood et al. (17)
	24	M	65	22.3	Splenectomy	2	Died at 2 months	Kazemimood et al. (17)
	25	M	51	8.5	Splenectomy	19	No recurrence	Kazemimood et al. (17)
	26	M	68	2.3	Splenectomy	6	No recurrence	Kazemimood et al. (17)
	27	F	51	5.3	Splenectomy	5	No recurrence	Kazemimood et al. (17)
	28	M	67	7.5	Splenectomy	5	No recurrence	Kazemimood et al. (17)
	29	F	52	9	Splenectomy	12	No recurrence	Kazemimood et al. (17)
	30	F	67	6	Splenectomy	12	No recurrence	Jin et al. (18)
	31	F	77	5.6	Splenectomy	NA	NA	Jin et al. (18)
	32	F	71	4.5	Splenectomy	6	No recurrence	Chen and Yu (19)
	33	F	68	3.5	Splenectomy	NA	NA	Eid et al. (20)
	34	M	54	10.0	Splenectomy	9	No recurrence	
	35	M	65	5.5	Splenectomy	19	No recurrence	
Liver	1	F	68	12.0	Left hepatectomy	30	No recurrence	Selves et al. (21)
	2	F	35	20.0	Right hepatectomy	95	Recurrence	Shek et al. (22)
	3	M	37	15.0	Right trisegmectomy + S1 resection	42	No recurrence	Twh et al. (23)
	4	F	57	9.5	Refusion	36	Alive	Chen, Kuo, and Ng (24)
	5	F	51	1.7	Left hepatectomy	12	No recurrence	Chen, Kuo, and Ng (24)
	6	F	19	12.0	Tumorectomy	40	No recurrence	Cheuk et al. (8)
	7	F	56	15.0	Right hepatectomy	56	Recurrence	Cheuk et al. (8)
	8	F	40	12.5	Left hepatectomy	108	Recurrence	Cheuk et al. (8)
	9	F	49	4.2	Tumorectomy	9	No recurrence	Cheuk et al. (8)
	10	F	31	15.0	Right hepatectomy	60	No recurrence	Cheuk et al. (8)
	11	M	82	10.0	Right hepatectomy	18	No recurrence	Ulises et al. (25)
	12	F	30	5.6	Right hepatectomy	24	No recurrence	Bai et al. (26)
	13	M	65	5.8	NA	2	Died at 2 months	Kazemimood et al. (17)
	14	F	28	1.5	Tumorectomy	NA	No recurrence	Xiaoyu et al. (27)
	15	F	57	NA	Tumorectomy	24	No recurrence	Granados et al. (28)
	16	M	40	NA	Tumorectomy	3	No recurrence	Zhengxiang et al. (29)
	17	F	59	6.0	Tumorectomy	17	No recurrence	Qing et al. (30)
	18	M	75	NA	Tumorectomy	6	No recurrence	Shuhong et al. (31)
	19	F	54	10.0	Tumorectomy	13	Died at 13 months	Pardasani et al. (32)
	20	F	53	11.5	Left hepatectomy	6	No recurrence	Paulo et al. (33)
	21	F	54	1.3	Tumorectomy	12	No recurrence	Shen et al. (34)
	22	F	28	6.0	Left hepatectomy	48	No recurrence	Yanyang et al. (16)
	23	F	48	23.3	Extended right hemihepatectomy	23	No recurrence	Yanyang et al. (16)
	24	M	60	3.0	Wedge resection	3	No recurrence	Yanyang et al. (16)
	25	F	19	6.0	Segementectomy	12	No recurrence	Zhang et al. (35)
	26	F	67	4.0	Radical right hepatectomy	NA	No recurrence	Deng and Gao (36)
	27	F	48	10.0	Right hepatectomy	2	No recurrence	Zhang et al. (37)
	28	F	31	3.5	Laparoscopic right hepatectomy	10	No recurrence	Zhang et al. (37)
	29	F	60	4.0	Laparoscopic left radical hepatectomy	12	No recurrence	Abe et al. (38)
	30	M	53	11.1	Middle hepatectomy	43	Recurrence	

^*^The maximum diameter of the resected specimen measured during pathological examination. NA, not available; F, female; M, male.

The pathogenesis of this tumor is closely associated with chronic EBV infection, likely attributable to EBV’s tropism for B lymphocytes and its latent genes contributing to tumorigenesis through apoptosis inhibition and immune evasion (38, 39). Specifically, EBV drives malignant transformation of follicular dendritic cells via latent membrane protein 1 (LMP1)-mediated hyperactivation of the nuclear factor kappa B (NF-κB) signaling pathway, resulting in pathological hallmarks including spindle-to-ovoid tumor cells arranged in fascicles or storiform patterns, peritumoral lymphocytic infiltration, thin-walled vascular proliferation, and focal necrosis/hemorrhage. The presence of EBER *in situ* hybridization positivity can strongly suggest EBV infection within the tumor and is crucial for the diagnosis of EBV+ IFDCS.

Patients with EBV+ IFDCS usually lack clinical symptoms; therefore, radiology examinations are often the first method for detection. The imaging manifestations directly and indirectly reflect the aforementioned pathological features: CT scans of all three patients revealed poorly demarcated, solitary soft tissue masses in our case series, exhibiting heterogeneous density due to necrosis/hemorrhage, demonstrating patchy heterogeneous enhancement (marked enhancement in vascular-rich areas and non-enhancement in necrotic zones); MRI T2-weighted imaging (T2WI) exhibits mixed high-low signal intensities corresponding to cellular-dense regions, collagen fibers, and hemosiderin deposition, while T1-weighted imaging (T1WI) may show focal hyperintensity related to hemorrhage (40). Crucially, EBV+ IFDCS not only presents common radiological characteristics but also manifests distinct organ-specific imaging phenotypes shaped by tissue microenvironment heterogeneity. Hepatic lesions manifest on CT as hypodense masses with central non-enhancing area, pathologically corresponding to LMP1-induced vascular hyalinization and thrombotic ischemic necrosis via excessive vascular endothelial growth factor (VEGF) pathway activation (41). Conversely, splenic lesions demonstrate “snowflake-like” T2WI hyperintensity with delayed enhancement on MRI, reflecting an inflammatory-tumor microenvironment characterized by EBV-driven lymphocyte infiltration and microhemorrhages. These organ-specific imaging variations not only unveil EBV’s divergent molecular mechanisms (vascular dysregulation vs. inflammatory recruitment) but also provide critical diagnostic discriminators. For instance, hepatic EBV+ IFDCS must be differentiated from hepatocellular carcinoma, which typically arises in cirrhotic livers and displays “wash-out” enhancement patterns, and from angiosarcoma, which exhibits aggressive features such as central patchy persistent enhancement with infiltrative margins (42). In the spleen, EBV+ IFDCS requires distinction from sclerosing angiomatoid nodular transformation (SANT), characterized by well-demarcated centripetal progressive enhancement and spoke-wheel patterns, and from splenic lymphoma, which often presents as multifocal coalescing masses with mild enhancement, splenic hilar/retroperitoneal lymphadenopathy, and restricted diffusion on diffusion weighted imaging (DWI).

The heterogeneous nature of microenvironments imparts organ-specific prognostic influences. Our findings demonstrate that patients with primary EBV+IFDCS originating in the liver exhibit a mortality rate nearly twice that of patients with splenic tumors during follow-up. Specifically, within the liver microenvironment, liver sinusoidal endothelial cells (LSECs) exhibit elevated expression of VEGF and intercellular cell adhesion molecule-1 (ICAM-1), facilitating tumor cell adhesion and angiogenesis (43). Concurrently, Kupffer cells (KCs) secrete IL-6 and TGF-β, thereby inducing epithelial-mesenchymal transition (EMT) in tumor cells. In contrast, the splenic microenvironment demonstrates a higher density of CD8+ T cells and NK cells within the red pulp, which suppress tumor proliferation through the PD-1/PD-L1 axis (44). Additionally, splenic tumor cells show heightened susceptibility to ferroptosis due to hypoxic conditions, whereas the oxygen-rich hepatic microenvironment promotes tumor survival (43). These prognostic disparities highlight the necessity for rigorous clinical management strategies in primary hepatic EBV+IFDCS to optimize patient outcomes.

In recent years, as more cases of a similar nature have been documented, our comprehension of EBV+ IFDCS has enhanced. Overall, the three cases presented in this study are consistent with the existing literature pertaining to this disease. When intra-abdominal lesions resembling inflammatory pseudotumor, particularly in the spleen, are suspected, diagnosing EBV+ IFDCS can become comparatively straightforward. Magnetic resonance imaging (MRI), with its high resolution, provides crucial detailed information about the lesion, aiding in a more precise diagnosis.

## Conclusion

EBV+ IFDCS is an uncommon and frequently underrecognized neoplasm that can manifest in various anatomical locations. Its rarity, combined with non-specific clinical presentations and imaging characteristics that may resemble those of other inflammatory or neoplastic conditions, makes EBV+ IFDCS a significant challenge for differential diagnosis. Consequently, this leads to low preoperative diagnostic rates and a potential for misinterpretation.

This report details three cases of EBV+ IFDCS, presenting their clinicopathological characteristics and imaging features, including CT and MRI findings. By reviewing relevant literature, the study aims to deepen the understanding of EBV+ IFDCS, highlight key diagnostic indicators, and improve diagnostic accuracy and awareness among medical professionals. Ultimately, this work seeks to reduce the likelihood of misdiagnosis and guide more precise and timely therapeutic interventions.

## Data Availability

The original contributions presented in the study are included in the article/supplementary material. Further inquiries can be directed to the corresponding author.
