# Supplementary material for: EBV-positive inflammatory follicular dendritic cell sarcoma occurring in different organs: a case report and literature review
Source: Front Oncol. 2025 Jul 31;15:1603496. doi: 10.3389/fonc.2025.1603496 (PMC12351499; doi:10.3389/fonc.2025.1603496)
Supplement: Supplementary file 1 [file Table1.docx]

Table S1. Brief summary of the Clinicopathological Features of EBV-positive inflammatory follicular dendritic cell sarcoma from the Present Study and the Literature

| **Characteristics** | **Present study** | **Literature** | **Total** |
| --- | --- | --- | --- |
| **Total cases** (n) | 3 | 62 | 65 |
| **Median age** (year) (range) | 34(30-35) | 47(19-77) | 47(19-77) |
| **Sex** (female/male) | 3/0 | 39/23 | 42/23 |
| **Location** (splenic/hepatosplenic) | 2/1 | 33/29 | 35/30 |
| **Recurrence** (yes/no/NA) | 1/2/0 | 6/50/6 | 7/52/6 |
| **Median follow-up** (month) | 19 | 10 | 10 |
| **Outcome** (died/alive) | 0/3 | 3/59 | 3/62 |

NA: not available.
